# Removal of Aggregates During Bispecific Antibody Purification Using Hydrophobic Interaction Chromatography

**DOI:** 10.3390/membranes15100299

**Published:** 2025-10-01

**Authors:** Puya Zhao, Yue Qi, Kai Gao

**Affiliations:** Shanghai AsymChem Biotechnology Co., Ltd., Building 8, No.12, Lane 855, Jinzheng Road, Jinshan Industrial Park, Shanghai 201507, China; gaokai@asymchem.com.cn

**Keywords:** downstream process, membrane chromatography, quality by design, flow-through mode

## Abstract

In the production of recombinant antibody/Fc-fusion proteins using mammalian cells, many aggregates often form alongside the target proteins, particularly with bispecific antibodies. To ensure the safety of biological products, it is essential to control the amount of aggregates within a specific range. A traditional downstream process typically involves using Protein A (ProA) resin to capture the target antibody, followed by two polishing steps to ensure purity; for instance, using an anion exchange chromatography (AEX) in flow-through mode and a cation exchange chromatography (CEX) in binding–elution mode. In this study, we choose a Dual Action Fab (DAF), which can bind two antigens and is prone to aggregation when expression in CHO (Chinese Hamster Ovary) cells. We introduce hydrophobic interaction membrane chromatography (HIMC) operating in flow-through mode, which enhances production efficiency while reducing costs and the risks associated with column packing. We evaluated the impact of the operating buffer system, as well as the pH and conductivity of the loading samples, on aggregate removal using HIMC. Additionally, we investigated the mechanism of aggregate binding and found that loading conditions had a limited impact on this process. Overall, our findings indicate that employing HIMC can achieve a 20% reduction in aggregate levels. These results demonstrate that HIMC in flow-through mode is an effective and robust approach for reducing aggregates during antibody purification.

## 1. Introduction

Bispecific antibodies (BsAbs), engineered to simultaneously engage two distinct epitopes on a single target or across different molecular entities, have emerged as transformative therapeutic modalities since their conceptual inception [1,2]. The evolutionary trajectory of BsAbs traces back to the 1960s, when pioneering studies demonstrated the feasibility of generating bispecific F(ab’)2 molecules through re-association of antigen-binding fragments derived from heterologous polyclonal sera. A paradigm shift occurred with Köhler and Milstein’s hybridoma breakthrough in 1975, enabling systematic exploration of rodent monoclonal antibody conjugation and hybridoma fusion technologies to achieve precise dual-targeting specificity [3]. Subsequent decades witnessed an exponential diversification of BsAb architectures, culminating in accelerated clinical translation. The recent surge in therapeutic BsAb development reflects their distinct pharmacological advantages over conventional monoclonal antibodies, including augmented target engagement precision, enhanced tumor microenvironment penetration, improved manufacturability profiles, and reduced immunogenic risks—attributes that position BsAbs as potent weapons in modern immuno-oncology [4,5]. Nevertheless, the inherent structural complexity of these molecules poses unique challenges in downstream processing, particularly regarding aggregate removal during purification—a critical quality attribute demanding innovative chromatographic optimization strategies.

Bispecific antibodies (BsAbs) in modern bio-therapeutic development are predominantly produced through recombinant DNA technologies, having evolved into multiple structural formats that broadly categorize into two classes: IgG-like constructs with functional Fc domains and non-IgG-like frameworks lacking Fc-mediated effector functions [1,2]. While IgG-like architectures dominate clinical pipelines due to their favorable pharmacokinetic profiles, their complex heterotetrametric structure—particularly in asymmetric variants requiring coordinated assembly of two distinct heavy chains (HCs) and light chains (LCs)—presents substantial manufacturing challenges [6,7]. During the expression of BsAbs, aggregates may form due to structural instability or miss-folding of amino acid sequences. Environmental factors such as temperature fluctuations, suboptimal pH conditions, and improper ionic strength during production or storage can further exacerbate aggregation phenomena [8,9]. These aggregates pose significant challenges to therapeutic applications: Aggregates may sterically hinder antigen-binding domains, diminishing target recognition efficacy and compromising therapeutic outcomes [10]. Particulate aggregates can enhance immunogenicity by activating dendritic cells and triggering anti-drug antibody responses, potentially leading to hypersensitivity reactions or reduced drug persistence [11]. Aggregation accelerates physical degradation pathways, jeopardizing product integrity during long-term storage and transportation [12,13].

The rapid advancement of BsAb therapeutics necessitates parallel innovations in downstream purification strategies to address unique product-related impurities that conventional mAb processes cannot effectively resolve. Traditional polishing approaches relying on ion exchange chromatography (IEX)–whether through anion exchange (AEX) flow-through (FT) or cation exchange (CEX) binding–elution(BE) modes–exploit isoelectric point (pI) differences between target molecules and impurities [14]. However, these methods face significant limitations in BsAb purification, particularly in resolving critical impurities like hole–hole homodimers that exhibit nearly identical pI values to the desired product. This challenge has driven the exploration of alternative separation mechanisms, with hydrophobic interaction chromatography (HIC) emerging as a promising orthogonal approach [15,16]. HIC leverages subtle differences in surface hydrophobicity between species, operating through either FT or BE modes, where protein–resin interactions are modulated by salt concentration gradients. While HIC has established utility in antibody purification, conventional implementations face inherent limitations, including constrained binding capacity, suboptimal throughput, and potential risks of protein denaturation due to strongly hydrophobic ligands—challenges that become particularly acute in industrial-scale BsAb manufacturing where both product quality and process efficiency are paramount [17]. These constraints underscore the need for innovative HIC optimization strategies to realize their full potential in next-generation BsAb purification platforms.

In pursuing next-generation purification technologies, researchers prioritize developing cost-effective and high-efficiency chromatography platforms. Notably, microporous membrane chromatography demonstrates transformative potential in addressing inherent limitations of conventional packed-bed systems, including excessive media/operational costs, diffusion-dominated intraparticle mass transfer mechanisms, and scalability challenges related to column packing consistency [18,19]. The structural innovations of membrane chromatography drive multi-level technical advancements: Modular configuration eliminates column packing processes and associated variability. Tortuosity-free flow paths reduce pressure drops by 2–3 orders of magnitude compared to packed beds. Convection-enhanced mass transfer enables 10–50× higher linear flow rates without compromising binding capacity, dramatically accelerating adsorption–desorption kinetics [19,20,21,22,23]. The Sartobind^®^ Phenyl hydrophobic membrane adsorber, a pioneering innovation introduced, revolutionized biomolecule purification through its unique design [24]. The system integrates 30-layer radial flow capsules with an optimized 8 mm bed height, engineered to maximize operational efficiency. This breakthrough technology combines a specifically engineered macroporous membrane architecture with covalently immobilized phenyl ligands, creating a robust platform for high-throughput purification [25,26]. The membrane’s structural design enables exceptional flow rate tolerance while maintaining high binding capacities, addressing critical limitations of traditional resin-based systems. Its hydrophilic stabilized regenerated cellulose matrix provides superior mechanical stability and chemical resistance, even under stringent cleaning conditions. Notably, the membrane demonstrates minimal nonspecific protein binding—a critical advantage when processing high-concentration biomolecules in lyotropic salt solutions. Consistent with HIC principles, the functionalized membrane exhibits a characteristic increase in binding capacity with rising ammonium sulfate concentrations. This predictable behavior ensures reliable process scalability and simplifies method development across diverse biomolecule purification applications.

In downstream biopharmaceutical purification processes, both BE and FT modes represent widely employed chromatographic techniques, though their applications differ significantly based on resin characteristics [27,28]. The FT mode is predominantly utilized in AEX to remove residuals like host cell protein (HCP) and host cell DNA (HCD) rather than aggregates. The BE mode finds principal application in HIC and CEX, where target molecule–resin interactions require controlled binding and subsequent elution to get rid of aggregates. However, it is well known that the BE mode of HIC has a lower dynamic binding capacity (DBC) and requires an extended processing time and various buffers [29]. Additionally, the HIC-BE mode necessitates substantial salt addition to elevate sample conductivity during loading, which may induce protein precipitation and/or denaturation, potentially compromising product integrity [30]. On the contrary, the FT mode has a substantially higher loading density, adsorbs only impurities, and requires no additional recovery equipment [14]. Therefore, in this study, we will explore the possibilities of HIC membranes for both BE and FT modes to improve the process efficiency as much as possible while ensuring the removal of impurities.

Given these considerations, combined Sartobind^®^ phenyl membrane adsorber and FT mode, the efficiency can be greatly improved while removing the aggregates. This HIMC combines the advantages of HIC and membrane chromatography, ensuring no diffusional limitations, a shorter processing time, a high capacity, and excellent resolution [24]. When synergized with HIMC under convective flow regimes, this approach demonstrates transformative advantages. In this study, we evaluated the efficacy of CEX for aggregate removal, while comparatively investigating BE and FT modes in HIMC. The HIMC-FT mode demonstrated superior performance in aggregate clearance. Furthermore, a DOE approach was implemented to optimize loading conditions, capacity, recovery, and SEC purity, successfully establishing the optimal operational window.

## 2. Materials and Methods

### 2.1. Antibody and Its Properties

Chinese hamster ovary (CHO) glutamine synthetase knockout (GS−/−) host cell (HD-BIOP3, Horizon) were used to express recombinant antibodies with standard cell culture methods (stirred bioreactor) [31]. Cells were cultivated in batch mode: in 2 L Biostat^®^ bioreactors (Sartorius Stedim Biotech, Göttingen, Germany) for 14 days. A Sarto-clear BT 1000 filter with a pore size of 0.22 µm (Sartorius Stedim Biotech) was used to filter the cells after undergoing two-step centrifugation. The antibody employed in this experiment was a BsAb and its properties are listed in Table 1.

### 2.2. Equipment

This experiment was carried out using the Cytiva ÄKTA Avant 150 chromatography system. The system comprises two positive displacement pumps (A and B) connected to the mixing chamber. The flow was routed via the mixing chamber to the column. The UNICORNTM (version 7.6) software (Cytiva, Uppsala, Sweden) was used to operate the pumps and collect and process the data.

### 2.3. Protein A Chromatography

MabSelect SuRe LX (Cytiva, Uppsala, Sweden) was used to capture antibodies from harvested cell culture fluid (HCCF). The loading capacity was 30.0 g/L in accordance with the volume of the resin. The retention time was set to 5 min, and samples for analysis ranged from the UV_280_ peak of 50 mAu/mm to the maximum and then to 50 mAu/mm. Chromatography was carried out with the buffers, retention time (RT), and procedure volume listed in Table 2.

### 2.4. AEX

AEX was carried out with POROS 50HQ resin (Thermo Fisher, Bedford, TX, USA). The loading capacity was 150 g/L. The retention time was set to 5 min. Chromatography was carried out with the buffers and procedures listed in Table 3.

### 2.5. CEX

CEX was carried out with Capto S ImpAct resin (Cytiva, Uppsala, Sweden). The loading capacity was 40 g/L. The retention time was set to 5 min. The elution buffer was a 0–100% gradient combination of A: 50 mM NaAc-HAc, pH5.5 and B: 50 mM NaAc-HAc, 500 mM NaCl, pH5.5. Chromatography was carried out with the buffers and procedures listed in Table 4.

### 2.6. HIMC

Sartobind^®^ Phenyl (Sartorius Stedim, Göttingen, Germany) was a membrane used in the flow-through mode of the Hydrophobic Interaction Membrane Chromatography (HIMC), which has a volume of 3 mL, 8 mm bed height. Prior to the experiment, the loading sample’s conductivity and pH were adjusted in accordance with the equilibration buffer. The resin’s loading capability was 15 g/L. The fraction was collected between 50 mAu/mm and 100 mAu/mm, and the flow-through product was examined using SEC-HPLC. The BE mode (binding–elution mode) was also applied for evaluation purposes, chromatography was carried out with the buffers and procedures listed in Table 5. And the FT mode chromatography was carried out with the buffers and procedures listed in Table 6.

### 2.7. Analytical Techniques

#### 2.7.1. Determination of DBC

The binding capacity studies were carried out as follows: the membranes were initially cleansed with equilibration buffer for 15 membrane volumes (MVs) at the RT of 18 s. The HIMC Load (pH 5.5, 140 mS/cm, the sample conductivity was adjusted by 3 M (NH_4_)_2_SO_4_ solution) was then continuously loaded on Sartobind^®^ Phenyl with a membrane volume of 3 mL at a residence time of 18 s until the UV absorbance at 280 nm showed a 10% breakthrough. The concentration of the flow-through samples was measured using UV Spectrophotometer-NanoDrop one (Thermo Fisher, Delaware, DE, USA) with the molecule-specific extinction coefficients. No dilution was required for any of the samples. After that, the membrane was washed with 15 MVs of equilibration buffer, and the proteins were flushed out with 15 MVs of elution buffer. After stripping, the membrane was sanitized with 15 MVs of sodium hydroxide, neutralized with HPW, and stored in 20% *v*/*v* ethanol.

The amount of protein loaded into the membrane was calculated from the following equation:

Protein binding capacity = C_0_ × (V_L_ − V_0_)/V_M_, (1)

Note: C_0_—protein concentration in the sample (mg/mL), V_L_—accumulated volume per fraction (mL), V_0_—system void volume (mL), V_M_—membrane volume (mL).

#### 2.7.2. Design of Experiment (DoE) Screening Study Design and Data Analysis

**Figure 1:**
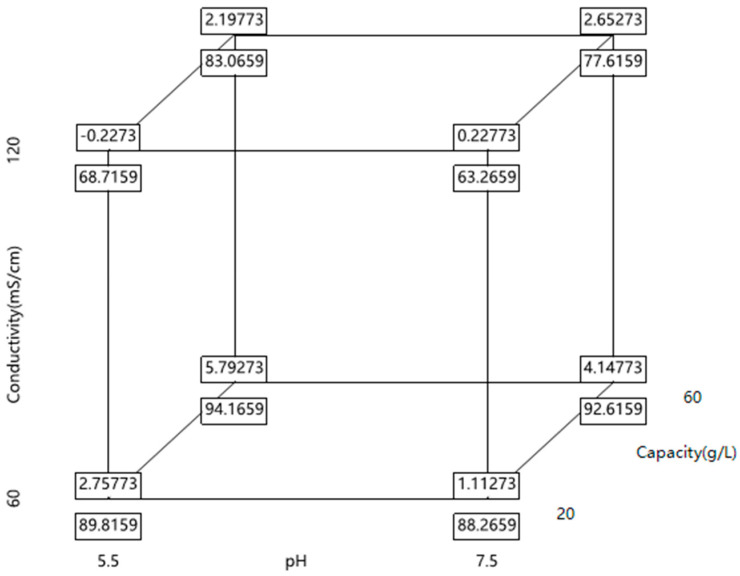
Schematic representation of the conducted design of experiments for the conductivity, pH, and capacity.

The conductivity and pH of the applied sample, along with loading capacity, were the three factors at two levels in the multivariate analysis. A complete factorial design was used to examine these three factors, requiring 8 experiments. Additionally, 3 center point experiments were added to improve accurate estimation of experimental errors and ensure that there are (sufficient) degrees of freedom to analyze experimental errors [32]. Since the complete factorial design allows for all interactions and linear effects, each factor may be assessed independently. The experimental setup is displayed in Figure 1 and Table 7. Optimal factor settings for the process were obtained by entering the findings of each experimental point into JMP^®^ (18) software after the optimization trial series was finished. Statistical examination of the generated data was part of the review process.

#### 2.7.3. Recovery

Spectrophotometric analysis was used to determine the BsAb concentration. Product recovery was calculated from Equation (2):

Recovery = mass of BsAb(monomers) in elution/mass of BsAb(monomers) applied to column or membrane(2)

#### 2.7.4. Size Exclusion Chromatography-High-Performance Liquid Chromatography (SEC-HPLC)

Agilent 1100 Series was applied as the SEC-HPLC equipment, and absorbance at 280 nm was used to quantify the amounts of monomers and aggregates. Samples were injected using 200 mM potassium phosphate, which contains 250 mM potassium chloride (pH 6.8) as the running buffer into a SEC column (250 Å, 5 μm, 7.8 mm × 300 mm, Waters, Taunton, MA, USA). The working flow rate was set to 0.8 mL/min. By combining the peak regions of the early-eluting aggregate peak(s), late-eluting fragment peak(s), and monomer peak, the ratios of monomers and aggregates were calculated, which indicated the aggregate levels of the product.

## 3. Results and Discussion

### 3.1. The HMWs Removal Ability Evaluation of the CEX Column

**Figure 2:**
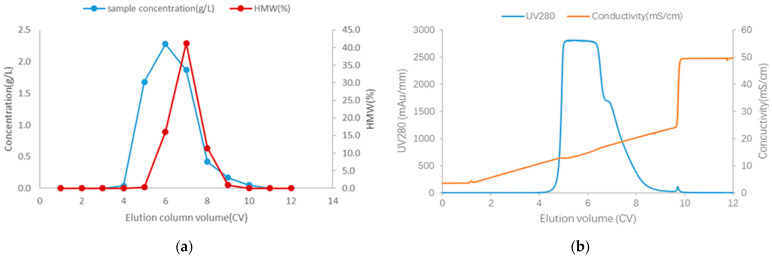
(**a**) CEX elution profile; (**b**) CEX UV280 adsorption and conductivity chromatography in the elution phase. Both showed a poor resolution between aggregates and monomers.

CEX and HIC are common methods to separate monomers and aggregates in antibody industry production. Compared with HIC, CEX has a lower requirement for loading conductivity and higher capacity [29,33]. Thus, the CEX (Capto S Impact, Cytiva) column was primarily tested to separate aggregates and monomers. The loading condition of CEX is pH5.5, conductivity 4.0 mS/cm, and the capacity is 30.0 g/L. Figure 2 shows the CEX profile in the elution phase. Based on Figure 2, the fraction eluate was CV 4–9. However, CV 6–8 contained HMW levels over 10.0%. Figure 2b shows a shoulder peak in the fraction eluate CV7. SEC-HPLC results of CEX load and fraction 4–7 collection are shown in Table 8. Thus, the resolution between aggregates and monomers was poor. Combined with Figure 2 and Table 8, it was challenging to separate HMWs and monomers based on the difference in pI between aggregates and monomers by the change in elution conductivity in CEX. Thus, HIC would be tested based on the hydrophobicity difference between monomers and aggregates.

### 3.2. The Evaluation of FT and BE Mode of HIMC

Compared with column chromatography, membrane chromatography has an advantage of short residence time, which can decrease the single experiment run time and increase research efficiency [21]. Thus, HIMC (Sartobind^®^ Phenyl, 3 mL) was attempted to remove aggregates in this study. To evaluate the aggregates removal ability of HIMC fully, both BE and FT modes were investigated.

#### 3.2.1. The Dynamic Binding Capacity of HIMC for BE Mode

**Figure 3:**
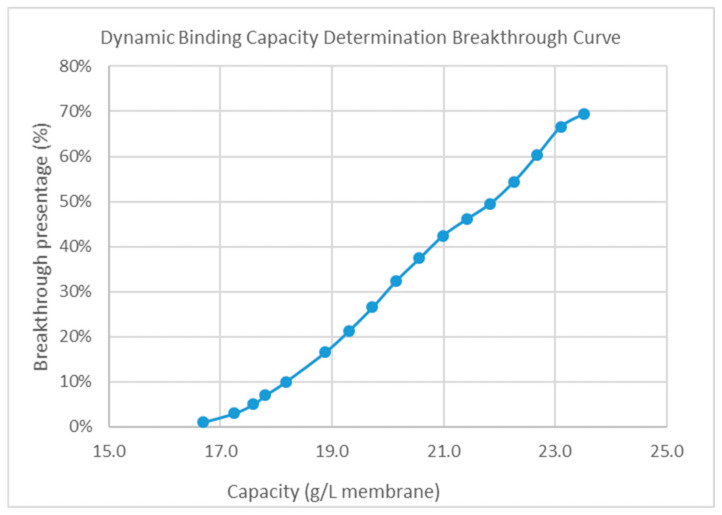
The dynamic binding capacity determination breakthrough curve of HIMC.

For HIMC BE mode, dynamic binding capacity (DBC) study was need to evaluate to find out the maximum loading density of the HIMC. To increase the binding capacity between sample and the ligand of HIMC, the loading conductivity should be large enough to increase the sample hydrophobicity. The loading condition of the HIMC was pH 5.50 and 140 mS/cm in this study. Figure 3 shows the DBC determination breakthrough curve at the RT 0.3 min. When the breakthrough was 10%, the corresponding DBC of HIMC was 18.2 g/L membrane. The final capacity of HIMC is DBC_10%_ × safety factor (0.8), thus, the capacity of HIMC in BE mode is 18.2 g/L × 0.8 = 14.5 g/L.

#### 3.2.2. The Aggregate Removal Ability of HIMC for BE Mode and FT Mode

**Figure 4:**
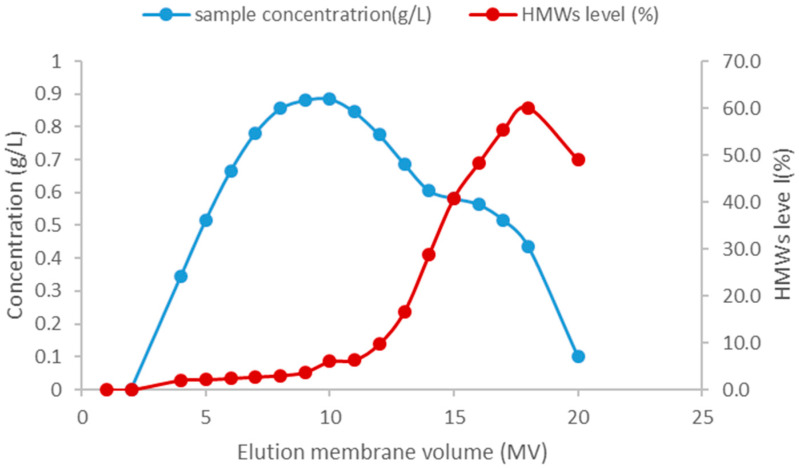
HIMC of BE mode elution profile.

**Figure 5:**
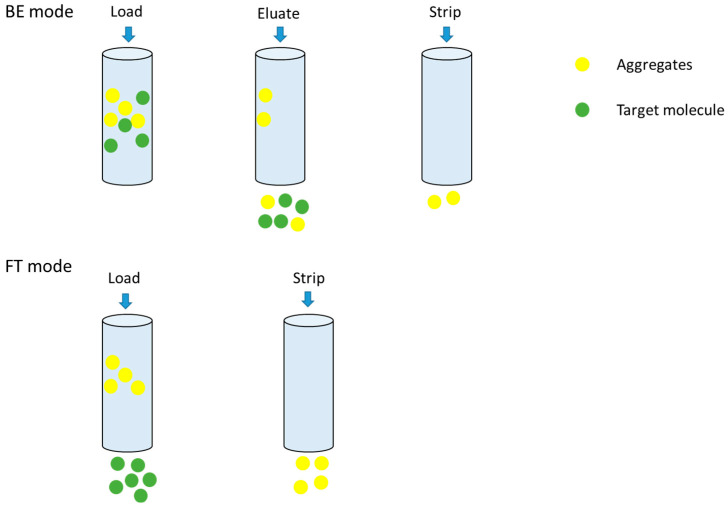
HIMC BE and FT mode working principle.

For the HIMC BE mode test, the loading density was 14.5 g/L, and the loading condition was pH5.5, 140 mS/cm. Based on previous research [34,35], the FT mode for column purification can tolerate a larger capacity than the BE mode, so for the initial attempt for the HIMC FT mode, the test conditions were the capacity of 30.0 g/L, and the loading condition was pH5.5, 90 mS/cm. The capacity and loading conditions are summarized in Table 9. Figure 4 shows the protein concentration, HMW level, and chromatography of HIMC BE mode in the Elution phase. Table 10 shows the recovery and SEC-HPLC purity for HIMC load, HIMC eluate, and FT pool. As shown in Table 8 and Table 10, although the capacity of HIMC BE mode is nearly half of the CEX BE mode and the recovery is less than 15.9%, the SEC monomers purity of HIMC BE mode is higher than that of CEX by about 14.6%. Compared with Figure 2, Figure 4 shows that HIMC BE mode has a higher resolution between aggregates and monomers than CEX BE mode. It shows that separation between aggregates and monomers by hydrophobicity is a potential method when the pI difference between monomers and aggregates is similar. The work principle of BE mode and FT mode of HIMC is shown in Figure 5. As shown in Table 10, HIMC FT mode has nearly twice its capacity, less 1.1% HMWs, and a higher 6.0% recovery than HIMC BE mode. It shows that HIMC FT mode has a higher potential than HIMC BE mode in the recovery, capacity, and aggregates removal ability section. HIMC FT mode optimization will be discussed in Section 3.3.

### 3.3. The Optimization of HIMC FT Mode by DoE Study and Analysis

#### 3.3.1. Experimental Strategy, Results, and Statistical Analysis of DoE

In the HIMC FT mode, aggregates can be attached to HIMC ligands by hydrophobicity interaction and monomers flow through directly. The loading density and condition, including pH and conductivity, may affect the attachment between aggregates and HIMC ligands. Thus, the DoE study was utilized to optimize the loading condition and loading density parameters. The DoE recovery and SEC-HPLC results are summarized in Table 11.

**Figure 6:**
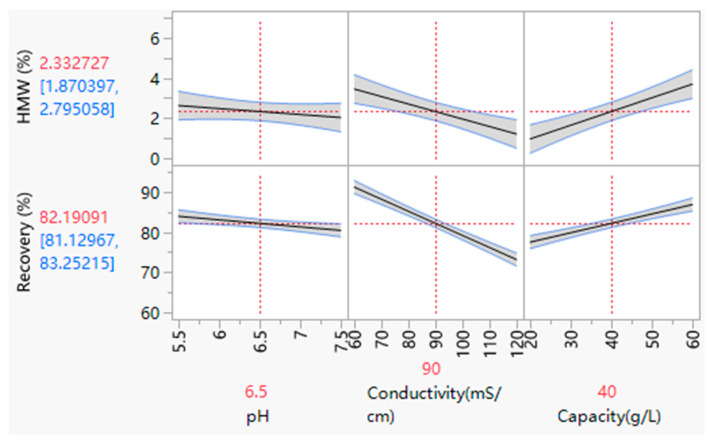
Prediction profiler depiction of changes in the HMW level and recovery as a function of the factors studied in the DoE. Note: vertical dotted lines represent the current value or current setting of each independent variable (X-variable); horizontal dotted lines indicate the current predicted value of each dependent variable (Y-variable) based on the current settings of the independent variables; the red fonts indicate the current value; the blue fonts represent the confidence intervals.

Statistical analysis was conducted on the responses of HMW level and recovery. JMP^®^ (18) software was applied for statistical analysis and a least squares fitting algorithm was used to obtain a good model for delta pressure of the HMW level (R^2^ = 0.94, model *p*-value = 0.0044) and recovery (R^2^ = 0.99, model *p*-value < 0.0001). The results of statistical analysis were shown in Figure 6. Further evaluation of the varied process parameter effects and interactions would be discussed in Section 3.3.3 and Section 3.3.4.

#### 3.3.2. The Load pH and Conductivity for HIMC FT Mode

As shown in Figure 5, the pH has a slight influence on HMW levels and recovery. However, the loading conductivity has a significant effect on HMW levels and recovery. With the loading conductivity increasing, the recovery will decrease, and the aggregates will also decrease in the HIMC FT pool. The reason may be that the high loading conductivity increases monomer and aggregates’ hydrophobicity interactions with HIMC ligands. With the strong interaction between aggregates and HIMC ligands, the HMW level will decrease in the FT pool. However, parts of monomers were attached with HIMC ligands, as well. As for loading pH, the increase in pH will slightly increase the monomers and aggregates’ hydrophobicity, especially if the loading conductivity is low (60 mS/cm), as shown in Table 11. If the loading conductivity increases, the pH variance influence of hydrophobic loading samples is weak. Thus, the load conductivity significantly influences HMW removal and recovery of HIMC FT mode more than the loading pH.

#### 3.3.3. The Capacity for HIMC FT Mode

The loading density also plays an essential role in HMW removal and recovery for HIMC FT mode, as shown in Figure 5. If the capacity increases, the aggregate removal ability will decrease, and the recovery of HIMC FT mode will increase. The reason may be that the binding sites between aggregates and HIMC ligands are in a specific range as the ligands of HIMC are limited. If the hydrophobicity of loading samples increases, the interaction between aggregates and HIMC ligands increases, and more aggregates can be attached to the same amount of HIMC ligands. Thus, increasing loading conductivity will increase the HIMC FT capacity without affecting recovery and HMW removal ability. Also, if the loading conductivity is so high, proteins may precipitate, resulting in protein loss. It is necessary for the protein to test conductivity adjuster tolerance before beginning the HIMC FT study.

#### 3.3.4. The Design Space of HIMC FT Mode

**Figure 7:**
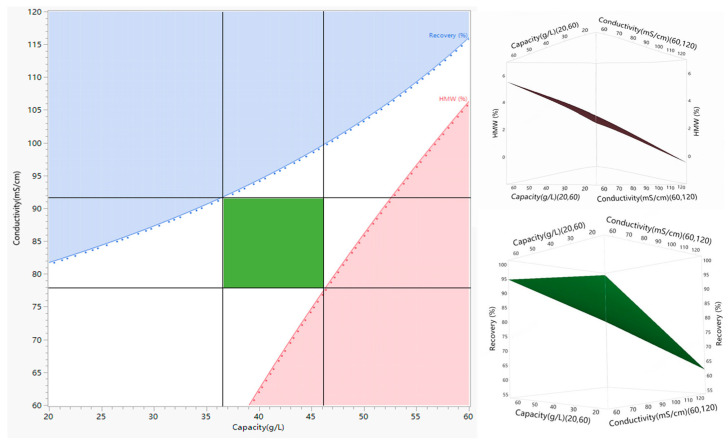
Design space based on the HMW level of less than 3.0% and recovery over 80.0%. The green area represents design space, the red area represents an HMW level of over 3.0%, and the blue area represents less than 80.0% recovery.

Based on the balance of recovery and HMW removal ability, the HMW level of less than 3.0% and recovery over 80.0% were set as the response requirements for HIMC FT mode. The design space was calculated by contour and surface plotters, as shown in Figure 7. Combined, the consideration of loading density and robustness of the process for scale-up, capacity between 37.0 and 45.0 g/L, loading pH 6.5, and loading conductivity between 78.0 and 91.0 mS/cm were recommended as the final process parameters.

## 4. Conclusions

In conclusion, this study investigates the aggregate removal ability for the CEX column and novel HIMC in both BE and FT modes. Compared with the traditional polishing step, CEX column purification, HIMC shows significant potential for separating aggregates from monomers, especially for complicated molecules, like BsAb. Furthermore, this study explores an innovative approach to the use of HIMC, the FT mode, which compensates for HIMC’s low load capacity and low resolution due to its own structural design. Compared to the HIMC BE mode, the FT mode has a higher aggregate removal ability. Meanwhile, the recovery and capacity of the HIMC FT mode are higher than that of BE mode. By HIMC FT mode, the HMW level can be decreased from 21.9% to less than 3.0% with a recovery of over 80.0%, which proves that HIMC FT mode is a robust and effective method for antibody purification production. This study only describes the removal of aggregates from a symmetric bsAb. Future research can focus on developing methods to remove aggregates from different types of molecules.

## Figures and Tables

**Table 1:** The antibody and its properties. The information includes the molecule class, pI and MW.

Molecule Class	pI	MW (kDa)
BsAb	8.15	150.52

Note: BsAb—bispecifc antibody, pI—Isoelectric point, MW—Molecular Weight.

**Table 2:** Procedure and buffers of protein A chromatography. There are experiment procedures, buffer and resistance time, and column volume for affinity chromatography.

Procedure	Buffer	RT (min)	Volume (CV)
Sanitization	1 M NaOH	5	3
Equilibration	50 mM Tris-HAc, 150 mM NaCl, pH 7.4		3
Elution	50 mM NaAc-HAc, pH 3.6		3
Regeneration	120 mM HAc		3

Note: CV—column volume.

**Table 3:** Procedure and Buffers of AEX. There are experiment procedures, buffer and resistance time, and column volume for AEX.

Procedure	Buffer	RT (min)	Volume (CV)
Sanitization	1 M NaOH	5	3
Equilibration	50 mM NaAc-HAc, pH 5.5		3
Strip	50 mM NaAc-HAc, 500 mM NaCl, pH 5.5		3

Note: CV—column volume.

**Table 4:** Procedure and buffers of CEX. There are experiment procedures, buffer and resistance time, and column volume for CEX.

Procedure	Buffer	RT (min)	Volume (CV)
Sanitization	1 M NaOH	5	3
Equilibration	50 mM NaAc-HAc, pH 5.5		3
Elution	A:50 mM NaAc-HAc, pH 5.5 B:50 mM NaAc-HAc, 500 mM NaCl, pH 5.5		0–100%B, 20 CV
Regeneration	50 mM NaAc-HAc, 500 mM NaCl, pH 5.5		3

Note: CV—column volume.

**Table 5:** Procedure and buffers of HIMC (BE mode). There are experiment procedures, buffer and resistance time, and column volume for HIC.

Procedure	Buffer	RT (min)	Volume (MV)
Sanitization	1 M NaOH	0.3	15
Equilibration	50 mM NaAc-HAc, 900 mM (NH_4_)_2_SO_4_, pH 5.5		15
Elution	A: 50 mM NaAc-HAc, 1.05 M (NH_4_)_2_SO_4_, pH 5.5 B: 50 mM NaAc-HAc, pH 5.5		0–100%B, 20 MV
Regeneration	HPW		15

Note: MV—membrane volume, HPW—high purity water.

**Table 6:** Procedure and buffers of HIMC (FT mode). There are experiment procedures, buffer and resistance time, and column volume for HIC.

Procedure	Buffer	RT (min)	Volume (MV)
Sanitization	1 M NaOH	0.3	15
Equilibration	50 mM NaAc-HAc, 450–900 mM(NH_4_)_2_SO_4_, pH 5.5		15
Regeneration	HPW		15

Note: MV—membrane volume, HPW—high purity water.

**Table 7:** Experimental study design. There are three factors: pH, conductivity and capacity.

Mode	pH	Conductivity (mS/cm)	Capacity (g/L)
0 0 0	6.5	90	40
0 0 0	6.5	90	40
− + −	5.5	120	20
− − +	5.5	60	60
+ − −	7.5	60	20
− − −	5.5	60	20
+ + −	7.5	120	20
+ + +	7.5	120	60
0 0 0	6.5	90	40
+ − +	7.5	60	60
− + +	5.5	120	60

Note: “0” represents the center point; “+” represents the high level; “−“ represents the low level.

**Table 8:** The quality data of CEX. The recovery and SEC-HPLC results of CEX load and collection eluate.

Sample	Recovery (%)	SEC-HPLC			CE-NR		HCP (ppm)	HCD (ppb)
		HMWs (%)	Monomers (%)	LMWs (%)	Purity (%)	LMWs (%)		
CEX load	N/A	20.9	79.1	ND	96.1	3.9	36.9	<0.3
CEX eluate 4–7	90.1	18.7	81.3	ND	96.2	3.8	3.6	<0.2

**Table 9:** The summary of capacity and loading condition of HIMC BE and FT mode.

Mode	Capacity (g/L Membrane Volume)	Loading pH/Conductivity (mS/cm)
Binding–elution	14.5	5.5/140.0
Flow-through	30.0	5.5/70.0

**Table 10:** The quality data of HIMC. The recovery and SEC-HPLC results of HIMC load, collection eluate MV 4–14 in BE mode and collection FT in FT mode.

Sample	Recovery (%)	SEC-HPLC			CE-NR		HCP (ppm)	HCD (ppb)
		HMWs (%)	Monomers (%)	LMWs (%)	Purity (%)	LMWs (%)		
HIMC load	N/A	20.9	79.1	ND	96.1	3.9	36.9	<0.3
HIMC eluate 4–17	74.2	4.1	95.9	ND	96.1	3.9	3.1	<0.3
HIMC flow-through	80.2	3.0	97.0	ND	96.3	3.7	3.4	<0.3

**Table 11:** The quality data of DOE. The recovery and SEC-HPLC results of DoE.

Mode	pH	Conductivity (mS/cm)	Capacity (g/L)	HMW (%)	Recovery (%)
0 0 0	6.5	90	40	1.81	83.5
0 0 0	6.5	90	40	1.79	83.1
− + −	5.5	120	20	0.31	67.3
− − +	5.5	60	60	6.16	94.1
+ − −	7.5	60	20	1.48	88.2
− − −	5.5	60	20	2.80	89.5
+ + −	7.5	120	20	0.10	64.3
+ + +	7.5	120	60	3.19	76.2
0 0 0	6.5	90	40	1.76	81.5
+ − +	7.5	60	60	4.19	92.3
− + +	5.5	120	60	2.07	84.1

Note: “0” represents the center point; “+” represents the high level; “−“ represents the low level.

## Data Availability

The original contributions presented in this study are included in the article. Further inquiries can be directed to the corresponding authors.

## Abbreviations

The following abbreviations are used in this manuscript:

BsAbs	Bispecific antibodies
ProA	Protein A
DAF	Dual Action Fab
CHO	Chinese Hamster Ovary
AEX	Anion exchange chromatography
CEX	Cation exchange chromatography
HIMC	Hydrophobic interaction membrane chromatography
HCs	Heavy chains
LCs	Light chains
IEX	Ion exchange chromatography
PI	Isoelectric point
HIC	Hydrophobic interaction chromatography
mAbs	Monoclonal antibodies
MW	Molecular weight
BT	Binding–elution
FT	Flow-through
CV	Column volume
MV	Membrane volume
HPW	High-purity water
HMWs	High molecular weights
C_0_	Protein concentration in the sample (mg/mL)
V_L_	Accumulated volume per fraction (mL)
V_0_	System void volume (mL)
V_M_	Membrane volume (mL)
DoE	Design of experiment
SEC	Size exclusion chromatography
HPLC	High-performance liquid chromatography
HCD	Host cell DNA
HCP	Host cell protein
RT	Retention time
